# Reproductive skew in cooperative breeding: Environmental variability, antagonistic selection, choice, and control

**DOI:** 10.1002/ece3.5502

**Published:** 2019-09-04

**Authors:** Peter Nonacs

**Affiliations:** ^1^ Department of Ecology and Evolutionary Biology University of California Los Angeles CA USA

**Keywords:** cooperative breeding, inclusive fitness, reproductive skew

## Abstract

A multitude of factors may determine reproductive skew among cooperative breeders. One explanation, derived from inclusive fitness theory, is that groups can partition reproduction such that subordinates do at least as well as noncooperative solitary individuals. The majority of recent data, however, fails to support this prediction; possibly because inclusive fitness models cannot easily incorporate multiple factors simultaneously to predict skew. Notable omissions are antagonistic selection (across generations, genes will be in both dominant and subordinate bodies), constraints on the number of sites suitable for successful reproduction, choice in which group an individual might join, and within‐group control or suppression of competition. All of these factors and more are explored through agent‐based evolutionary simulations. The results suggest the primary drivers for the initial evolution of cooperative breeding may be a combination of limited suitable sites, choice across those sites, and parental manipulation of offspring into helping roles. Antagonistic selection may be important when subordinates are more frequent than dominants. Kinship matters, but its main effect may be in offspring being available for manipulation while unrelated individuals are not. The greater flexibility of evolutionary simulations allows the incorporation of species‐specific life histories and ecological constraints to better predict sociobiology.

## Graphical abstract

This work examines the evolution of cooperation through a genetic simulations that incorporate ecological and life history constraints. The results show how previously static inclusive fitness models can dynamically expand to consider more realistic conditions.

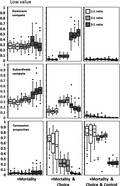

## 1 INTRODUCTION

Social groups often share the characteristic of unequal reproductive success across their members. This variance (i.e., reproductive skew) can reflect stochastic chance, but in many social settings random outcomes can be statistically rejected (Nonacs, 2000). If such skews are then deliberately created, why do a group's “losers” accept these outcomes? One possibility is that all win by gaining higher fitness than breeding solitarily (Vehrencamp, 1983). For example, whenever group productivity exceeds *nS* offspring (*n* = group members and *S* = mean number of offspring produced by a solitary individual), reproductive skews are possible in which all group members do better than by remaining alone (Nonacs & Hager, 2011). Thus, cooperation could reflect a mutually beneficial, but not always equal, social contract over the allocation of reproduction (Reeve & Nonacs, 1992).

Social contracts can be transactional (Reeve & Ratnieks, 1993), wherein group members allow or concede reproductive opportunities to induce joining or to maintain within‐group harmony. Concessions would not directly affect overall group productivity. Alternatively, individuals could contest for reproductive share which does reduce group productivity. In this case, the social contract is a compromise solution based on individual relative competitive ability (Reeve, Emlen, & Keller, 1998). However, mathematical models that consider only transactions or compromises often differ in predicting reproductive skew. To resolve this discrepancy, later treatments by Johnstone (2000) and Reeve and Shen (2006) incorporate both concessions and contests into a single, mathematical framework.

The synthesis model of the “bordered tug‐of‐war” (BTOW: Reeve & Shen, 2006) therefore predicts reproductive skew as determined by two behaviors: the willingness to peaceably concede a fraction of reproduction while simultaneously contesting the remainder. These behaviors occur in an ecological context of how successful solitary (noncooperative) individuals are and the amount of group‐level productivity that is gained through having additional group members. The BTOW can also incorporate individual differences in competitive effectiveness per unit of competitive investment, which then defines dominant and subordinate group members. Finally, to include gains of indirect fitness, the relatedness between group members need to be specified.

The basic premise of reproductive skew models like the BTOW is that subordinate behavior is an adaptive choice that increases fitness over noncooperation. This has not held up well to scrutiny, as a growing body of work finds that individuals likely pay a fitness cost for becoming subordinate helpers (Gadagkar, 2016; Kapheim, Nonacs, Smith, Wayne, & Wcislo, 2015; Nonacs, Liebert, & Starks, 2006; Rehan, Richards, Adams, & Schwarz, 2014; Shell & Rehan, 2018). Overall, models based on maximizing inclusive fitness have a poor track in matching prediction to observations of reproductive skew (Nonacs & Hager, 2011). One explanation for this is they are missing a number of potentially important elements. Six of these are as follows:

1. Antagonistic selection. A dominant parent may produce offspring that become subordinates and vice versa. Hence, advantageous behaviors when in one role may be disadvantageous in the other (Akçay & Van Cleve, 2016; Lehmann, Mullon, Akçay, & Cleve, 2016).
2. Population structure. Competitiveness within a group may increase reproductive share, but noncompetitive groups may be more productive overall. If such groups differentially contribute to population‐wide offspring production, then group selection could favor concessions and reduced competition (Reeve & Hölldobler, 2007; Wilson, 1975).
3. Environmental variability. Group sizes are likely to vary in populations. Subpopulations may inhabit areas of differing quality that change the value of subordinates and group productivity, and migration rates between subpopulations can vary. Also, cooperation can be destabilized by variance across group members in their willingness to share reproduction (Kokko, 2003).
4. Mortality. An individual may join a group as a subordinate, but if the dominant dies, it might be able to promote into that status. Reciprocally, if subordinates die then dominants can gain their contribution to group productivity without any concession or contest. The potential to gain direct fitness in this manner has been proposed as an explanation for why subordinates may initially tolerate unfavorable skews (Kokko & Johnstone, 1999; Leadbeater, Carruthers, Green, Rosser, & Field, 2011).
5. Choice. A prospective joiner may be able to choose among groups and then join the one that offers the highest expected fitness return (Grinsted & Field, 2017a, 2017b).
6. Control. Differences in competitive ability in the BTOW are assumed to be stochastic phenotypic features. However, dominants may be in position to impose a poorly competing phenotype on potential subordinates (e.g., Kapheim, Bernal, Smith, Nonacs, & Wcislo, 2011). If this capability is an evolvable entity, this could have large implications for reproductive skew.

Conceptually it is at least hypothetically possible that all six factors could be incorporated into an inclusive fitness equation. There have been attempts to expand skew models to examine a particular additional factor such as group size or competition between groups (e.g., Johnstone, Woodroffe, Cant, & Wright, 1999; Reeve & Emlen, 2000; Reeve & Hölldobler, 2007; Reeve & Jeanne, 2003). In practice, however, solving for optimal solutions rapidly becomes dauntingly complex. Consider Equation 5–7 for the optimal solutions from the BTOW (Reeve & Shen, 2006), which is the simplest case of determining reproductive shares between only two individuals. Furthermore, solutions have a strong game dynamic wherein one player's best strategy critically depends on its opponent's chosen strategy. Evolutionarily stable states (ESS's) can arise where no individual can either increase or decrease its level of competition to gain higher fitness. Very often, however, ESS solutions in the BTOW occur where group productivity has become less than *nS* (Nonacs, 2010). At this point, one or more group members ought to do better by leaving the group and reproducing solitarily. The models predict group dissolution rather than cohesion.

The alternative to an inclusive fitness approach is to consider the evolution of cooperation and reproductive skew as a problem in natural selection and through genetic simulations (e.g., Allen & Nowak, 2016; Kapheim et al., 2015; Nonacs, 2011; Nowak, McAvoy, Allen, & Wilson, 2017; Nowak, Tarnita, & Wilson, 2010). With such an approach the effects and interactions of the above six factors can be determined and a more complete picture of reproductive skew in nature can emerge.

## 2 METHODS

### 2.1 General framework of the model

A single simulation run follows the genetic evolution of a stable population of 480 agents for 5,000 generations. Each new generation is the offspring of the previous generation. Individual genomes have a gene locus for competitive effort as a dominant individual (*x*) in the group, a locus for competitive effort as a subordinate (*y*), and a third locus expressed in dominants as a reproductive concession (*p*) to subordinates. A given simulation starts with all individuals having *x* randomly drawn from a range of 0–0.2 (i.e., a less competitive starting population) or a range of 0.3–0.5 (a more competitive starting population). Similarly, *y* values are also drawn from either one of the two same ranges. Low concession populations are drawn from a 0–0.3 range, while high concession populations are drawn from a 0.5–0.8 range. In total, this produces eight combinations of *x*,* y*, and *p* starting ranges. Every generation of the simulation, each allele has 0.02 chance of mutation. If mutated, an allele's value is increased or decreased by a randomly chosen value between ±0.05; given the constraints of remaining within 0 and 1 bounds. Mutation allows for particularly advantageous alleles to reappear if stochastically eliminated when rare at the beginning of simulations.

ANOVA tested for significant effects on outcomes with starting conditions as the independent variables. Each combination is replicated four times for 32 total simulations for each defined scenario (see below). Programs are coded in TrueBasic™ and a copy is available in Appendix S1.

The first individual assigned to a site becomes the dominant, and subsequent joiners are subordinates. This means that dominant status is a purely phenotypic trait, independent of individual genotypes or parental status. This would be similar to dominance in nature reflecting an arbitrary character such as size or condition that follows from fortuitously being in the upper part of a distribution in provisioning effort. Alternatively, this could reflect a pattern often observed in wasps where dominant status is best explained by a “convention,” whereby the first female to start building the nest will be dominant to all that join her (Nonacs, 2001; Seppa, Queller, & Strassmann, 2002).

### 2.2 Determining reproductive success

The model follows the BTOW formulation of reproductive skew for determining an individual's expected reproductive success. The realized group output, summed across all group members, is a function of the potential maximum added productivity, *f*(*G*), that a given number of subordinates (*n*) can provide in a specific habitat, decremented by the level of within‐group competition, or:

(1) $$f\left(G\right)\left(1-x-by_{\text{max}}\right)\geq 0$$

where *y*_max_ is the effort the single most competitive subordinate devotes to activities that otherwise could have been invested into group‐level productivity. The *b* term weights the degree to which subordinates effectively compete against dominants (0 ≤ *b* ≤ 1). The use of *y*_max_ to represent subordinate competition creates an evolutionary trade‐off for subordinate behavior. More competitive individuals gain greater shares from between‐subordinate competition (see below) but at the cost of reducing overall group productivity. “Competition” could encompass a variety of tactics from aggressively fighting for reproductive opportunity to being passively “lazy” and withholding effort to take advantage of reproductive opportunity.

The gain function from having subordinates follows the relationship:

(2) $$f\left(G\right)=h\left(1+\frac{1}{2^{1}}+\frac{1}{2^{2}}+\ldots +\frac{1}{2^{\left(n-1\right)}}\right)$$

For a given simulation, adding subordinates would provide either low, medium or high benefits in terms of potentially increasing group productivity (average *h* = 0.5, 1.5, or 2.5, respectively). All gain functions assume that each subordinate adds less to overall group productivity (Nonacs, 1991). In this case, each additional subordinate adds 50% of what the previous joiner added. All solitary individuals receive a baseline fitness value set to an arbitrary value of *S* = 1 and dominant individuals retain this baseline if joined. Thus, the model assumes that larger groups always have potentially higher total productivity than smaller groups, and the dominant group member will never do worse than being solitary. This obviates the need to consider situations where the dominant might aggressively oppose being joined. It is important to note, however, that it is mathematically possible with higher *h* values for dominants to concede productivity gains to their subordinates such that they have a per capita fitness equal to or lower than a subordinate.

The dominant's share of the nonconceded added reproduction from having subordinates is proportional to its intrinsic willingness to compete relative to the entire group's total competiveness:

(3) $$\left(1-p\right)x/\left(x+b\sum_{n}^{1}y_{i}\right)$$

Each subordinate's share of the nonconceded reproduction is similarly:

(4) $$\left(1-p\right)by_{i}/\left(x+b\sum_{n}^{1}y_{i}\right)$$

All subordinates share in the reproduction conceded to them by the dominant relative to their intrinsic willingness to devote effort into competition (*y_i_*). Therefore, each subordinate's share of the conceded reproduction is:

(5) $$py_{i}/\sum_{n}^{1}y_{i}$$

The total fitness or reproductive share for individuals is as follows. For solitary individuals it is *S*; for dominants, it is the product of 1 and 3 plus *S*; and for subordinates, it is the product of 1 and (4 + 5). Each individual's fitness is then converted into a relative value within the population it is competing with for parentage. Parents are randomly chosen from this distribution, with the bias that individuals with greater relative fitness are more likely to be drawn.

This model differs slightly from the BTOW in that the BTOW assumes a possible reciprocal concession of reproduction from subordinates to dominants (*q*). Here, subordinate concession can be considered as not contesting the dominant's intrinsic gain separate from any benefits from cooperation. This varies dynamically but is always such that the absolute amount is constant across all simulations, or 1 = *qf*(*G*) = *S*. Also unless stated otherwise, *b* always equals one in all model scenarios (i.e., all individuals are intrinsically competitively equal per unit investment in competition). Intuitively, if parents imposed *b* < 1 on their own dispersing offspring, they would likely be making offspring that are easily outcompeted by other parents' more robust offspring.

The fundamental difference from the BTOW is that its solutions follow from an inclusive fitness maximizing process. This requires specifying the kinship between dominants and subordinates. This model instead follows the evolution of hypothetical gene loci. Hence relatedness need not be explicitly defined, but instead can evolve as a byproduct—that is, selection for specific trait values results in a population mostly sharing those values and thus being highly genetically similar at those loci as if related by recent descent. Also, the BTOW solutions are for pairwise interactions only, while the model here allows for consideration of groups with multiple and varying numbers of subordinates.

For the first 2,500 generations, reproduction is sexual in that each offspring has two parents with its genotype is drawn by random at each locus from one of the two chosen parents, and all subpopulations have the same *h* value. This mimics each trait being on a separate chromosome and allows selection to operate on each trait independently relative to mean benefit that helping provides. For the last 2,500 generations, reproduction switches to clonal where a single parent contributes an offspring's entire genome as if the traits are linked on the same chromosome. Also (except in the One vs. Two Gene Pool scenario), rather than each subpopulation having the same value of *h*, for the last 2,500 generations *h*'s are randomly drawn from a gamma probability distribution around the mean so as to include a more realistic variation in habitat quality across areas (Figure S1). Thus, even with a global mean of *h* = 0.5, some subpopulations are still productive enough that it is possible for both dominant and subordinate fitness to exceed that of being solitary. For greater values of *h*, most subpopulations can at least potentially have skews where most or all subordinates do better than being alone (Figure S1). Overall, this allows for selection for the best overall combination of traits, given that across generations genomes will find themselves in both dominant and subordinate bodies and in areas where helping provides more or less benefit.

### 2.3 Model scenario: one versus two gene pools

The BTOW model considers optimal investment strategies within a generation, without any consideration of antagonistic selection across generations. Mimicking this situation a model variant has separate gene pools for dominants and subordinates such that dominants can only have dominant parents and subordinates can only have subordinate parents. Therefore, *x, y,* and *p* loci are always in the “right” type of body for their optimal expression. Populations consist of either 240 groups of two or 96 groups of five. With separate gene pools, the first individual in a group is, therefore, drawn from the pool of dominant offspring and all subsequent group members are drawn from the pool of subordinate offspring. The one‐pool simulation variant differs only in having a single pool of potential parents such that dominant parents can produce subordinate offspring and vice versa.

### 2.4 Model scenario: environmental variability

It is unlikely that natural populations would be composed of entirely 2 or 5 individual groups. Therefore, this scenario and subsequent ones divide the total population into 12 subpopulations of 40 individuals each. In a given simulation run, all the subpopulations have either 40, 20, or 8 sites suitable for reproduction, producing population‐wide individual to site ratios of 1:1, 2:1, or 5:1, respectively. Within a subpopulation, all individuals are randomly assigned to sites. Therefore, although the modal group size is either 2 or 5, there can be considerable variation around those values. Reproduction is biased to occur within subpopulations, with three levels of movement or migration between subpopulations having means of 1, 4, or 20 immigrants per generation. Added to the three levels of *h*, this gives 27 unique combinations of subordinate value, individual to site ratio, and subpopulation mixing.

### 2.5 Model scenario: variability plus mortality

Should the dominant group member die, in many species the top‐ranked subordinate takes its place. Thus, this scenario adds to the above a 10% chance of any group member dying. If the dominant does die, the first subordinate to join promotes to dominant. Given that this subordinate is chosen randomly, there is no selection possible in the model to be the top‐ranked subordinate. Should group members die, the group retains their contributions to overall productivity as (*f*(*G*)) is determined by the original number of subordinates. The deceased individual is no longer party to how reproductive shares are allocated and its *y* value is dropped from calculating ((3), (4), (5)). Therefore, the expected fitness of being a subordinate is boosted by a nonzero chance of becoming dominant. Conversely, a dominant has a nonzero chance to gain the added productivity from a subordinate without having to concede or contest any of that additional benefit. Similarly, with multiple subordinates, the death of one creates the potential for surviving subordinates to claim a portion of the added reproduction.

### 2.6 Model scenario: variability plus mortality plus choice across sites

This variant is the same as above, but adds the ability of each individual to choose between three randomly selected groups. They join the group that gives the highest relative fitness as calculated from ((1), (2), (3), (4), (5)) based on the number of individuals already at the site and their genotypes. This creates a market dimension to joining decisions, as dominants are in competition to offer a potential subordinate more reproduction than a competing dominant or empty site (Grinsted & Field, 2017a, 2017b).

### 2.7 Model scenario: variability plus mortality plus choice plus control

This variant is the same as above except all individuals have an added gene locus that can exert dominant control over subordinates (the *b* value in Equations 3 and 4). Simulations start with individuals being assigned a *b* value randomly drawn from between 0 and 1. A value of *b = *0 means dominants can unilaterally render subordinates competitively incapable of taking any nonconceded reproduction away from the dominant. The BTOW model assumes that *b* is intrinsic to a subordinate's genome, but it seems improbable that selection would favor mutations for reducing one's own competitive ability. Thus, *b* is modeled here as a trait expressed in dominants that impose diminished competitive ability on individuals choosing to act as subordinates. This locus is transmitted, selected and potentially mutates as described for the other three.

### 2.8 Model scenarios: parent–offspring kinship

Groups are initially formed as in the Environmental Variability scenario, but then either 50% or 100% of the subordinates are replaced by offspring of the dominant (the “mother”) and a second parent is randomly chosen from within the subpopulation to act as the “father”.

### 2.9 Model scenario: parent–offspring kinship plus control

This variant is the same as 100% Kinship except all individuals also have a selectable *b* locus. Evolved control equates to the possibility that parental manipulation can bias offspring life history into subservient cooperation (Alexander, 1974; Kapheim et al., 2015). Coercive maternal rearing strategies have been strongly implicated in why some daughters remain as workers in a halictid bee (Kapheim et al., 2011).

## 3 RESULTS

### 3.1 One versus two gene pools

**Figure 1:**
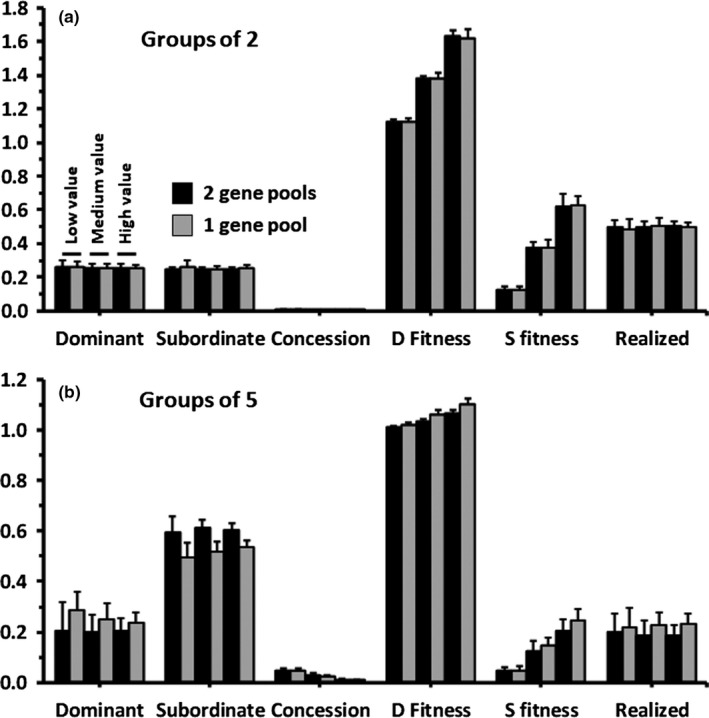
Two gene pools versus one. (a) All groups are composed of one dominant and one subordinate. The groups of six bars represent increasing *h* values of 0.5, 1.5, and 2.5. Mean values (with *SD*s) are given for dominant (*x*) and subordinate (*y*) competitiveness, proportional concession of reproduction to subordinates (*p*(*f*(*G*) − 1)), dominant and subordinate fitness, and the proportion of the total value subordinates could add to group productivity that is realized and not lost to competition. Fitness values greater than one indicate that, on average, individuals do better in groups than being solitary. (b) Same as (a), except that all groups have five members

Confirming Nonacs's (2010) 2‐player game approach for the BTOW, concessions are never given by dominants regardless of group size (Figure 1). For the two gene pool scenario: (a) Variation in the value that subordinates provide (*h*) increases fitness, but has no effect on ESS values of *x, y,* or *p*; (b) Both *x* and *y* are insensitive to differences in *h,* and (c) Overall within‐group competitiveness evolves to values such that approximately (1−1/*n*)*f*(*G*) of potential gain is lost due to competition.

Whether individuals reproduce in separate gene pools or the same one produces no difference in outcomes when group size is two (Figure 1a). When group size is five, however, mean *x* and *y* values are consistently either larger or smaller in the single gene pool than the separate pools (Figure 1b: ranging in magnitude of 10%–40%). To further examine this effect, another simulation was run with a 20:1 ratio and *h* = 4.5. The differences in predicted *x* and *y* values become consistently greater; on the order of 25%–50%.

There is no consistent effect from the starting conditions at the beginning of a simulation run for *x, y,* and *p* values.

### 3.2 Environmental variability

**Figure 2:**
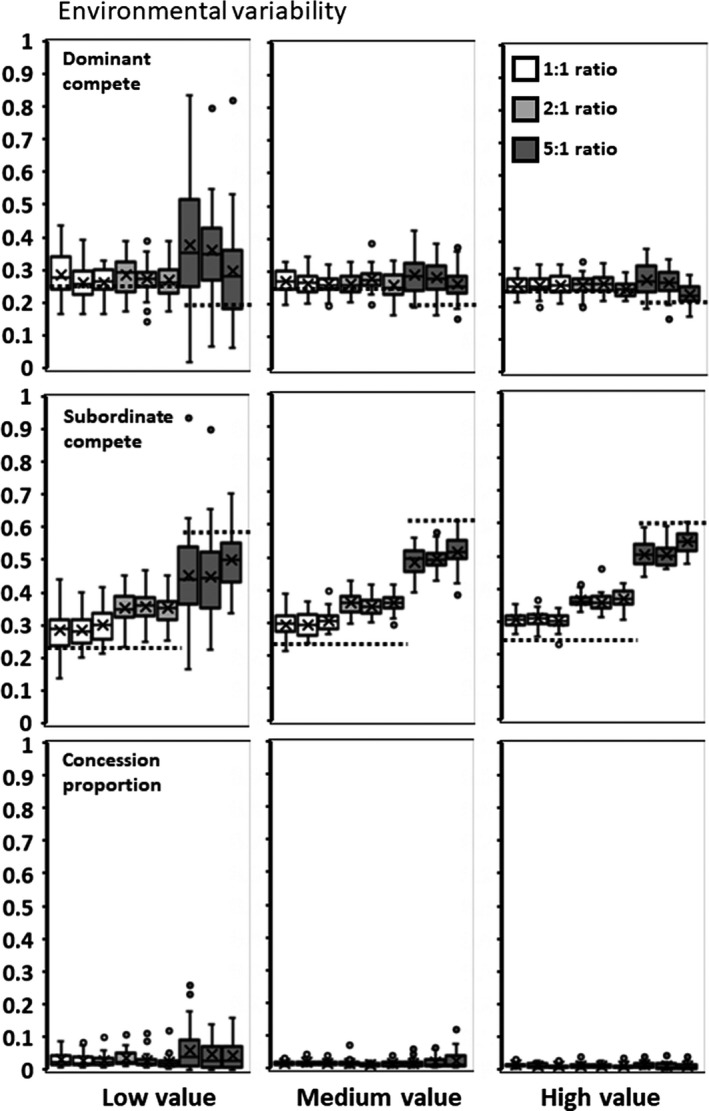
Environmental variability. Top row of panels are values for dominant competitiveness (*x*). Middle row are values for subordinate competitiveness (*y*). Bottom row are values for the proportion of realized productivity that dominants concede to subordinates (*p*(*f*(*G*) − 1)). Columns are for the value of adding subordinates (*h*). Shading groupings denote the ratio of individuals to suitable site. Within a shaded group, migration rates vary from a mean of 1 per generation to 10% or 50% per generation. The *X* is the distribution mean. Dotted lines are for comparison of *x* and *y* values from the two gene pools scenario for groups of 2 or 5

Mean dominant competitiveness (*x*) is similar across both ratios of individuals to sites and value added by subordinates (*h*) (Figure 2). Mean subordinate competitiveness (*y*) increases with ratio, but is similar across *h*. Dominants concede little to no reproduction to subordinates. The addition of environmental variability does not substantially alter the mean values of *x,* and *y* from those arising from the two gene pool scenario described above (and dotted lines in Figure 2). The largest deviations are in mean subordinate competitiveness.

The consistently high levels of *x* and *y* means that a considerable proportion of value subordinates could add to group productivity goes unrealized (Table 1). Subordinates, on average, never achieve fitness levels equal or exceeding the fitness of being a solitary individual (Table 1).

**Table 1:** The mean proportion of potential value from subordinates that is realized and the mean subordinate fitness

*h*	Ratio	Variation	+Mortality	+Choice	+Control	100% Kin	50% Kin	100% K + Control
Mean realized value from subordinate addition								
Low	1 and 1	0.429	0.429	0.865	0.958	0.704	0.567	0.964
Low	2 and 1	0.355	0.375	0.594	0.961	0.713	0.513	0.972
Low	5 and 1	0.186	0.192	0.292	0.977	0.600	0.345	0.976
Medium	1 and 1	0.427	0.428	0.702	0.973	0.714	0.551	0.979
Medium	2 and 1	0.362	0.367	0.478	0.983	0.711	0.503	0.984
Medium	5 and 1	0.197	0.203	0.194	0.987	0.616	0.351	0.985
High	1 and 1	0.422	0.433	0.583	0.983	0.711	0.554	0.984
High	2 and 1	0.358	0.371	0.387	0.986	0.699	0.499	0.986
High	5 and 1	0.195	0.204	0.166	0.987	0.611	0.347	0.985
Mean subordinate fitness								
Low	1 and 1	0.113	0.218	0.523	0.571	0.214	0.160	0.051
Low	2 and 1	0.090	0.168	0.340	0.454	0.218	0.148	0.033
Low	5 and 1	0.035	0.068	0.074	0.108	0.126	0.072	0.017
Medium	1 and 1	0.335	0.493	1.067	1.154	0.609	0.463	0.090
Medium	2 and 1	0.275	0.389	0.727	0.992	0.639	0.429	0.062
Medium	5 and 1	0.110	0.155	0.118	0.266	0.389	0.223	0.032
High	1 and 1	0.553	0.784	1.337	1.492	0.998	0.774	0.119
High	2 and 1	0.456	0.625	0.960	1.302	1.032	0.703	0.085
High	5 and 1	0.183	0.246	0.161	0.401	0.644	0.367	0.051

Note

Individual values are the average across all three levels of migration (they are, therefore, the mean of 1,152 subpopulations from 96 simulation runs of 5,000 generations each). There are three levels each of subordinate value (*h*) and individual to site ratios. Variation is the initial model to which Mortality, Choice and Control are sequentially added (see text). Subordinate fitness of less than one indicates that, on average, individuals would have attained higher fitness by being solitary.

**Figure 3:**
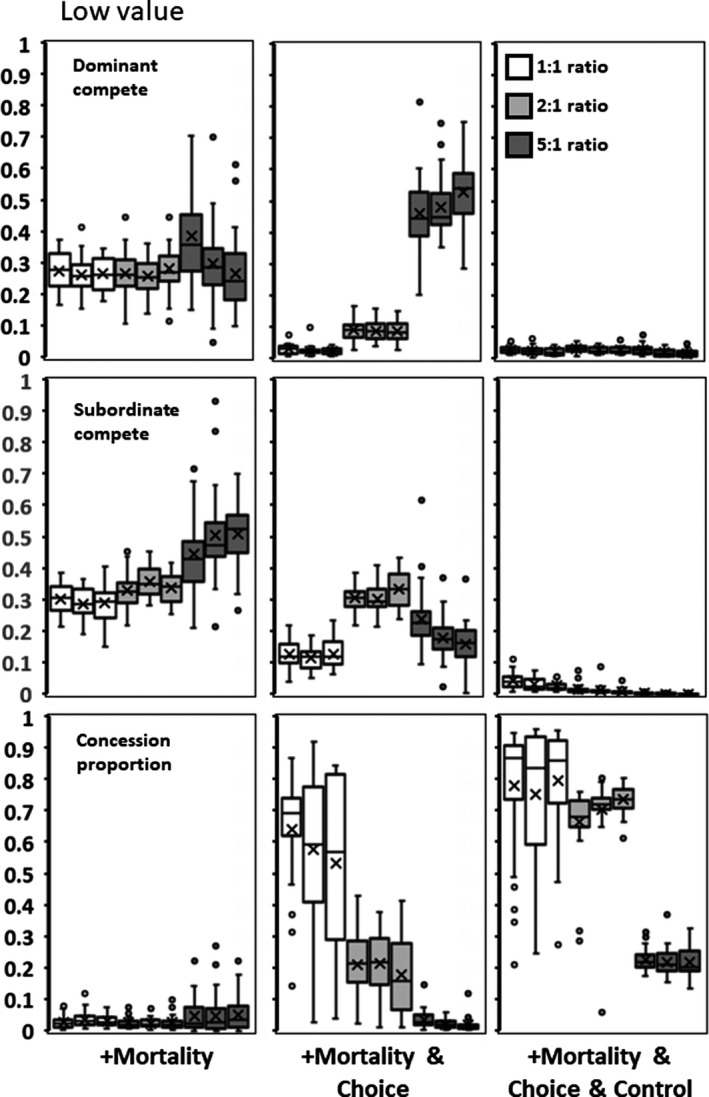
Low value for adding subordinates (*h*). Columns represent sequentially adding, in order from left to right: (1) Mortality to environmental variability; (2) Choice in which group to join; and (3) Dominant control over subordinate competitiveness. Rows are as in Figure 2

**Figure 4:**
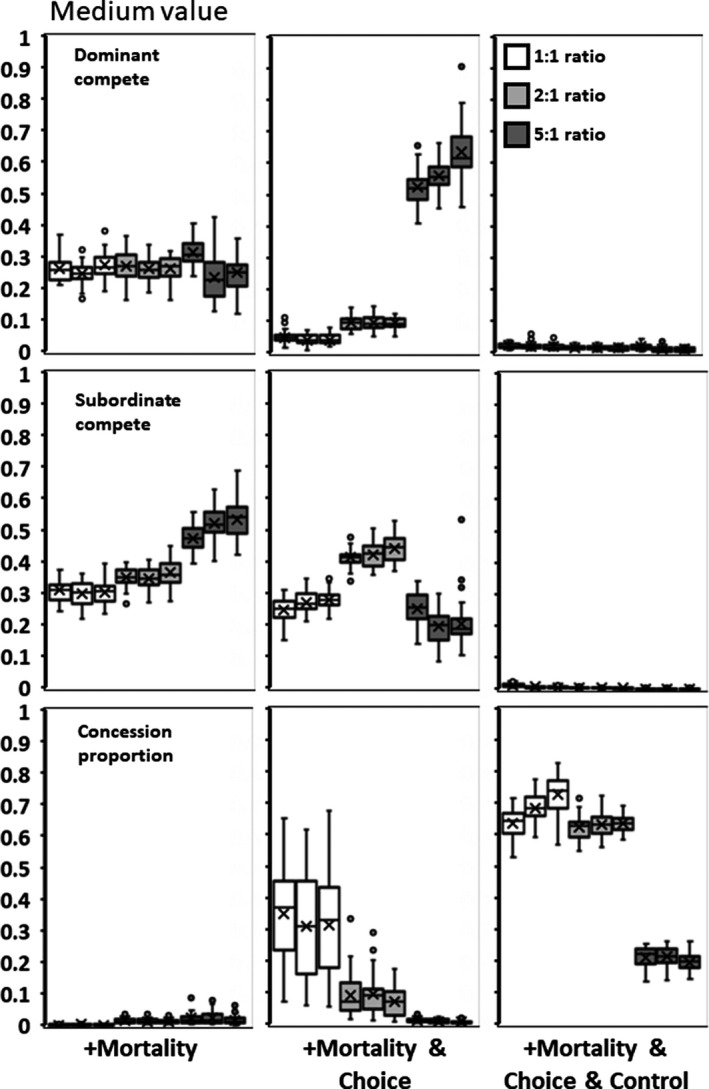
Medium value for adding subordinates (*h*). Panels as in Figure 3

**Figure 5:**
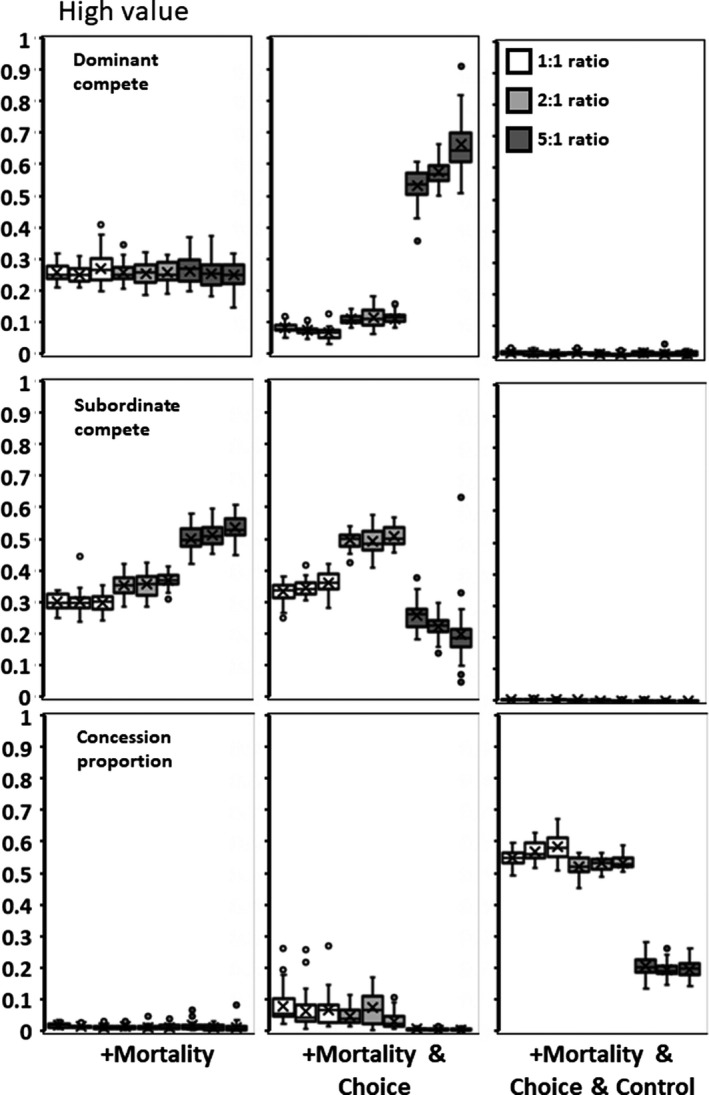
High value for adding subordinates (*h*). Panels as in Figure 3

**Figure 6:**
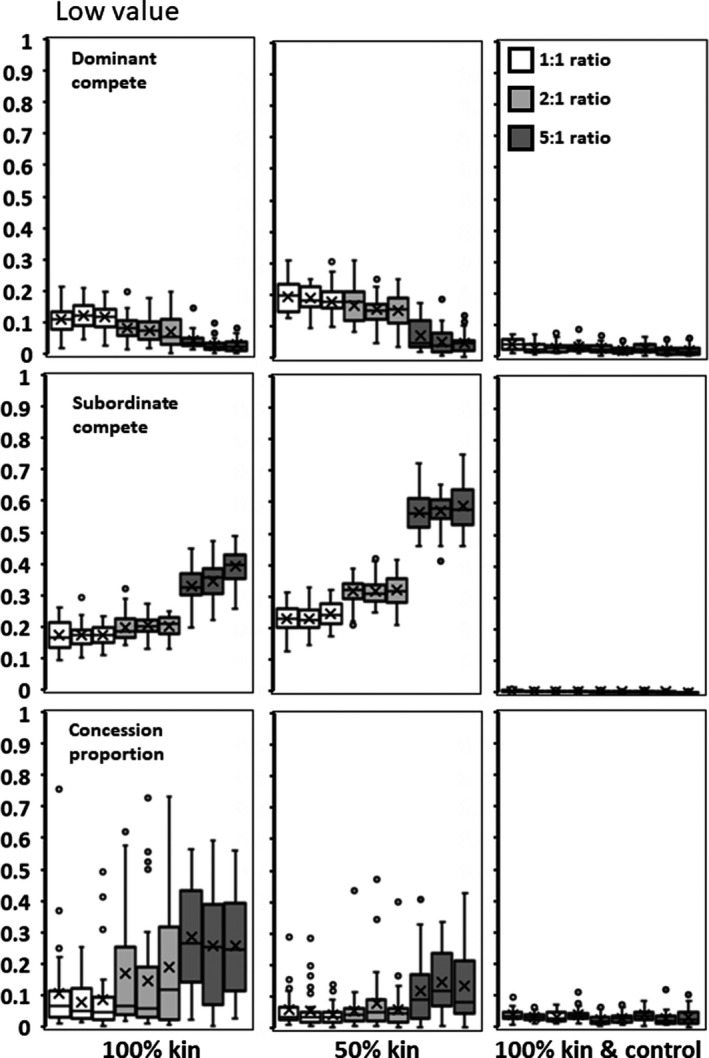
Low value for adding subordinates (*h*). Columns represent, in order from left to right: (1) All subordinates are offspring of the dominant; (2) 50% of subordinates are offspring of the dominant; and (3) All subordinates are offspring of the dominant and the dominant controls subordinate competitiveness. Rows are as in Figure 2

**Figure 7:**
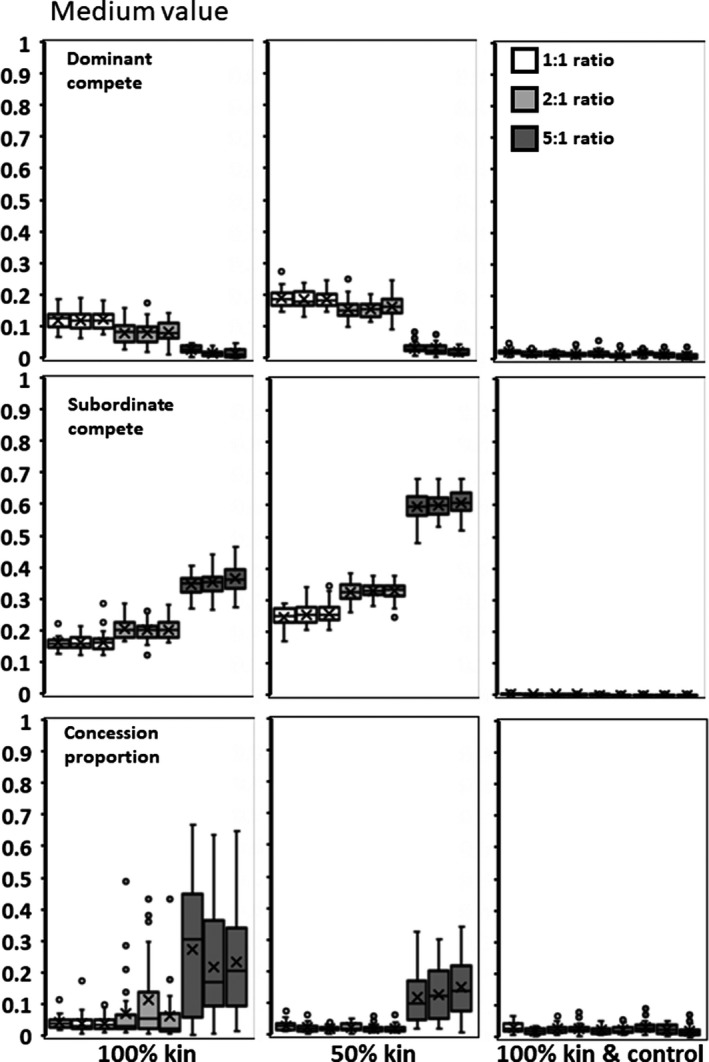
Medium value for adding subordinates (*h*). Panels as in Figure 6

**Figure 8:**
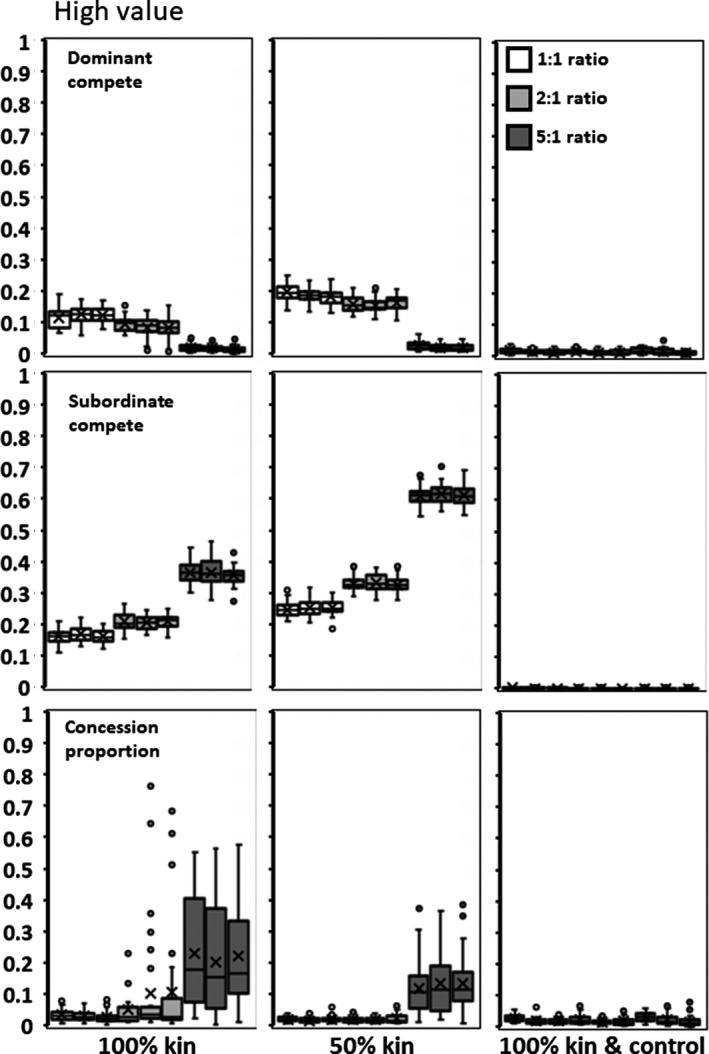
High value for adding subordinates (*h*). Panels as in Figure 6

For this scenario and all subsequent ones, there is no consistent effect on *x, y,* and *p* values from the rate at which individuals migrate between subpopulations (Figures 2, 3, 4, 5, 6, 7, 8).

When subordinates add low value, there is an effect of starting conditions for the 5:1 ratio (Figure 2, left column). Starting conditions of high versus low concession result in significant differences between the mean of *p* at the end of the simulations (0.068 vs. 0.032 for high and low, respectively: *F* = 25.506; *df* = 1,88; *p* < .0001). However, the increase in *p* does not significantly increase either the mean fitness of dominants and subordinates or the realized gain from having subordinates. Instead, individuals adjust by increasing their competitiveness (*x* and *y*) to offset any gains from the concession (Figure 2).

### 3.3 Variability plus mortality

The addition of a 10% mortality does not alter *x*,* y*, and concession outcomes in any substantive way from the above (compare Figures 3, 4, 5 to Figure 2). Although mean subordinate fitness now includes the occasional promotion to dominant, mean fitness levels never equal or exceed the fitness of being a solitary individual (Table 1). Starting conditions have the same effect as in above.

### 3.4 Variability plus mortality plus choice across sites

Allowing subordinates to choose between three sites to join has a large effect on outcomes. Dominant competitiveness is low with ratios of 1:1 or 2:1, but markedly increases with an individual to site ratio of 5:1 (Figures 3, 4, 5). Subordinate competitiveness declines at the lowest and highest ratios. Dominants make large concessions to attract subordinates when site ratios are less than 5:1. The combination of these values at lower ratios greatly increases the realized gain from having subordinates, and subordinate mean fitness is higher and can exceed that of a solitary individual (Table 1). These gains, however, disappear at the 5:1 ratio.

As found in the Environment Variability scenario, the initial values of *p* have effects on the final outcomes. A higher starting condition, for example, produces a mean concession of 0.873 in comparison to 0.857 for the 1:1 ratio (*F* = 31.367; *df* = 1,88; *p* < .0001, Figures 3, 4, 5, middle panels, bottom rows). In this case, groups did reap increased realized productivity (*F* = 3.784; *df* = 1,88; *p* = .0549), which was harvested to increase subordinate fitness (*F* = 5.248; *df* = 1,88; *p* = .0239).

### 3.5 Variability plus mortality plus choice plus control

The addition of a fourth genetic locus (*b*, the ability of dominants to suppress subordinate competitiveness) greatly affects outcomes (Figures 3, 4, 5). The control locus invariably evolves to be equal or close to zero, and therefore, mean subordinate competitiveness (*by*) is also always close to zero. Without having to compete against subordinates, dominants also evolve to have low levels of overt competition (*x*). This combination means that almost all the group‐level benefit that subordinates provide is directed into reproduction and not competition (Table 1). Interestingly, mean subordinate fitness also increases over the no control scenarios (and can be greater than being solitary) with the addition of dominant control (Table 1).

Higher starting conditions of *p* for produced mean concessions of 0.904 in comparison to 0.716 for the 1:1 ratio (*F* = 29.316; *df* = 1,88; *p* < .0001, Figures 3, 4, 5, right panels, bottom rows). In this case, groups did reap significantly increased realized productivity with more concessions (*F* = 8.216; *df* = 1,88; *p* = .0052). The gain distributes across both dominants and subordinates and, therefore, neither group showed a significant increase in mean fitness.

### 3.6 Parent–offspring kinship

When subordinates are all the offspring of the dominant, *x* and *y* decrease (Figures 6, 7, 8) relative to values in the Environmental Variability scenario. Groups, therefore, realize more of the potential productivity gains from having subordinates and mean subordinate fitness can exceed that of solitary individuals when subordinates provide high value (mean *h* = 2.5; Table 1). When only 50% of the members of a group are offspring of the dominant, then the values of *x*,* y*, and concession are intermediate between the 100% and Environmental Variability scenarios (Figures 6, 7, 8). This suggests that the proportion of kin in a group has a roughly linear effect on the genetics and the resultant group reproduction.

Higher starting conditions of *p* result in a higher final mean *p* of .592 compared to .297 for the 5:1 ratio with 100% kin (*F* = 38.396; *df* = 1,88; *p* < .0001). Although this creates variable amounts of concessions (Figures 6, 7, 8, left panels, bottom rows), groups do not realize significantly higher productivity based on initial condition. However, dominants do gain significantly increased fitness when they initially conceded less rather than more (*F* = 10.487; *df* = 1,88; *p* = .0017).

### 3.7 Parent–offspring kinship plus control

As found above, the control locus evolves to almost completely eliminate offspring ability to contest for reproduction (*b* = 0). With almost no within‐group competition as *x* and *y* are close to zero (Figures 6, 7, 8), groups realize almost 100% of the available productivity gains (Table 1). Dominants concede almost no reproduction (Figures 6, 7, 8), and therefore, subordinates produce almost none of the group's offspring (Table 1). Subordinate fitness is but a small fraction of what they would achieve as solitary individuals. The initial starting conditions had no significant effect.

## 4 DISCUSSION

The evolution of cooperative breeding and eusociality can reflect a complex interplay across ecology, behavior, and genetics. By application of an agent‐based evolutionary simulation, it becomes evident that the outcome of competition across group members for reproductive shares is most strongly affected by three factors: limits in the number of sites that allow for successful reproduction; the value added to group productivity by each subordinate member; and the potential to suppress within‐group competition. Behaviorally, it also matters in whether or not individuals can choose which group to join and if groups contain close kin or unrelated individuals.

The ESS solutions that arise under the majority of scenarios predict that the average subordinate has lower fitness than a solitary individual, and often considerably so (Table 1). This has an important implication for the evolution of cooperation. Without site limitations (i.e., if all individuals can always successfully reproduce by themselves), cooperative breeding evolves only when subordinates add large value to group productivity and group sizes are small. Site limitation, therefore, offers subordinates a best of bad choice between low fitness as a group member or zero fitness. Further, within‐group selection favors escalation until the ESS is reached, regardless of cost to group‐level success. The fitness ramifications of site limitation and the lack of outside options are particularly relevant for obligately social species (Creel, 1990; Lucas, Creel, & Waser, 1996) or in producing what appears to be obligate sociality (Strassmann, 1991).

Nevertheless, all group members could still potentially gain through cooperation if subordinates did add enough productivity value. For example, in the Environmental Variability scenario if *h* = 4.5, mean subordinate fitness can exceed solitary fitness (results not shown). Considering, however, that the first subordinate in a group would then need to add 450% to group success, it makes it also seem unlikely that such massive gains in group‐level success would be available in the initial evolutionary progression from a solitary to a social life history.

In contrast, if potential exists for dominants to manipulate or suppress subordinates into not competing, this markedly reduces how much group‐level productivity must increase. It is notable that unrelated subordinates always have higher fitness in groups where dominants have suppressed their ability to compete. This would suggest that group‐level selection might favor the evolution of self‐controlling, noncompeting genotypes. Although such group‐level effects were not detected here, they might still occur under scenarios not considered. For example, if the effects of competition on group productivity are nonlinear, predicted outcomes can significantly change (Nonacs & Hager, 2011).

As regards one's own offspring, parents can have the means to directly manipulate potential subordinates into adopting helper roles (Alexander, 1974; González‐Forero & Gavrilets, 2013; Kapheim et al., 2011, 2015). To the degree that it is genetically possible, control always evolves to completely suppress competition (Figures 6, 7, 8). This predicts that parent–offspring skew is nearly always total and that offspring are much more likely to be making the best of an enforced bad situation rather than willing collaborators.

The interactions between dominants and subordinates and the resulting group functioning is also strongly affected by subordinate choice. This creates a market economy in which dominants compete against each other to attract valuable subordinates (Grinsted & Field, 2017a, 2017b). A choice, as minimal as between only three groups, can force dominants to concede substantial amounts of reproduction and markedly reduce their competitiveness levels (Figures 3, 4, 5). However, this is only evident in the scenarios where the most frequent group size is two. When group sizes get larger (e.g., a mode of 5), the market flattens and choices between groups differ little in fitness gains. Perceptual limits for fitness differences may make differentiating between groups impossible and under such a constraint animal choice may revert to random (Cartar & Abrahams, 1997). Moreover, larger perceived differences by the first joiners will likely be erased as more individuals make their decisions. This was seen in high density, large group size populations of *Polistes dominula* where individuals switching between nests did not significantly improve their expected outcomes (Grinsted & Field, 2017b).

Finally, reproductive pattern produced different outcomes. When subordinates are the offspring of the dominant, rather than unrelated individuals, both dominants and subordinates are less aggressive and dominants concede more reproduction (Figures 6, 7, 8). Interestingly, that there are more concessions to kin than nonkin is the opposite of what most inclusive models predict (Nonacs & Hager, 2011). These results, however, are in the absence of an ability to control the subordinates' competitiveness. When given a possibility of manipulation and control, all concessions are withdrawn and parents produce close to 100% of the offspring (Figures 6, 7, 8).

The simulation results not only suggest factors that affect reproductive skews, but also identify factors that appear to have little to no effect. Foremost, antagonistic selection across generations is not explicitly considered in the BTOW model. When individuals differ predictably depending on age or role and frequency in the population then such antagonistic selection can be an important factor (Akçay & Van Cleve, 2016; Lehmann et al., 2016). However, treating dominants and subordinates as either contributing to the same or separate gene pools produces almost identical predictions for competitiveness and concession in groups of two (Figure 1a). Consistent differences do arise when groups have five or more members (Figure 1b). This is not surprising as the BTOW is explicitly derived for one dominant and one subordinate, and not for situations where subordinates simultaneously compete against both a dominant and other subordinates. Demographically this means that with ratios of individuals to sites greater than 1:1 the majority of surviving dominant offspring are likely to be subordinated in the next generation. Thus, as almost all reproductive skew models are based on pairwise interactions (Nonacs & Hager, 2011), they are likely to inaccurately predict behavior in species where multiple subordinates are common.

Subdividing the population into 12 smaller subpopulations and varying the level of migration between them produces no consistent differences in interaction patterns (Figures 2, 3, 4, 5, 6, 7, 8). Similarly, allowing group size and the value of subordinates across subpopulations to vary produces only minimal differences in subordinate competitiveness (Figure 2). Whether the initial population gave large or small concessions could produce alternative stable states in some scenarios. However, from the standpoint of affecting dominant or subordinate fitness, the effects are minimal. Overall group productivity is affected by initial conditions only when either choice or control is present. Subordinates having a choice results in higher fitness when populations are initially high conceding. Dominants having control results in their higher fitness when populations are initially low conceding. Starting conditions with kinship have no effect on overall group productivity, but low initial concession levels do increase dominant fitness. These results suggest that potentially intrinsic levels of interindividual aggressiveness or agreeableness in ancestral species may constrain to some degree the patterns of reproductive skew in the descendant social species. Finally, adding mortality into the simulations allows some subordinates to promote to dominant status and reap dominant‐level fitness. Although this increases the mean expected fitness for subordinates (Table 1), it does not significantly change levels of competitiveness or concessions (Figures 3, 4, 5).

## 5 CONCLUSIONS

Reproductive skew models assume that reproductive shares have two components: A portion of reproduction that is conceded without competition costs and the remainder that is competitively contested. In practice, it would be impossible to discriminate which offspring belong to which portion (Nonacs & Hager, 2011). Simulation results suggest, however, that measurable features of social groups such as overall productivity with subordinates, levels of competition, and the reproductive skew will provide testable predictions as to their underlying sociobiology. As examples:

- Societies in which dominant competitiveness levels are unaffected either by the value subordinates add to the group or by subordinate number predicts low within‐group relatedness and strong site limitations where individuals may have little choice in which groups they can join. This may apply in some species of ants where unrelated females band together to collaboratively initiate new colonies (Nonacs, 1989). For instance, in *Solenopsis invicta* individual investment is unaffected by the number of females in an association (Bernasconi & Keller, 1999).
- Where smaller groups exhibit lower competitiveness and reduced skew, but the dominant's competitiveness and reproductive share increase rapidly with group size also predicts strong site limitations along with some measure of choice in groups to join.
- Groups of all sizes and across all values of having subordinates in which there is little or no evidence of competition for reproduction, but subordinates have measureable reproductive success in the presence of dominants predicts a combination of choice in joining groups and some means of effective suppression or restraint of subordinate competition. In *Ceratina* bee species, dominance is settled without fighting and avoidance of direct competition may occur through egg eating. Subordinates do, however, reproduce a significant fraction of the offspring (Hogendoorn & Velthuis, 1999).
- Same as above, but with very little or no subordinate reproduction predicts that these groups are mostly composed of close kin rather than unrelated individuals. This appears to be the case in mother–daughter associations of *Megalopta genalis* where skews are high and little aggression is observed (Kapheim et al., 2015).
- Where dominant and subordinate competitiveness are declining and increasing functions of group size, respectively, predicts groups contain a significant percentage of close kin, but dominants cannot suppress or manipulate competitiveness. As the proportion of close kin in the group declines, reproductive skew should increase. Skew has been found to increase across a number of species (reviewed in Reeve & Keller, 1995) as the mean relatedness of the offspring produced decreases, but changes in the levels of competitiveness were not given.

Although reproductive skew has been measured across multiple species, suitable data to test the above predictions more fully is sparse and mostly suggestive. This reflects the past conceptual emphasis that existing models place on inclusive fitness and relatedness (*r*). There is also the fact that relatedness is often easier to measure or estimate than either effort devoted to competition, reproductive concessions, the level of control dominants can exert over subordinate reproduction, or the group‐level productivity value subordinates add. Thus, most existing tests are on the effects of differing levels of within‐group genetic relatedness (Nonacs & Hager, 2011). The advantage for agent‐based evolutionary simulations is that *r* need not be specified. It is either “built‐in” by whether groups form as collection of unrelated individuals or similar by descent, or “evolves‐in” as selection sweeps identical alleles to higher frequency or fixation.

What this approach uniquely reveals is that other factors, such as group size, the potential to suppress competition (particularly in relation to manipulating one's own offspring), and the ability to choose which group to join may all be more important for how cooperatively breeding societies arise than levels of relatedness. Evolutionary simulations are furthermore flexible enough to incorporate an even wider array of factors and life histories that might be present across species. To properly embrace and illuminate the complexity of cooperative behavior requires methods designed to exactly do that.

## CONFLICT OF INTEREST

The author has no conflict of interest.

## AUTHOR CONTRIBUTIONS

The author is solely responsible for all aspects of the work.

## Data Availability

There are no data to be archived.
