# Supplementary material for: Reproductive skew in cooperative breeding: Environmental variability, antagonistic selection, choice, and control
Source: Ecol Evol. 2019 Sep 4;9(18):10163–75. doi: 10.1002/ece3.5502 (PMC6787806; doi:10.1002/ece3.5502)
Supplement: Supplementary file 1 [file ECE3-9-10163-s001.docx]

**Appendix S1**

Gamma distributions for determining the values subordinates add.

**Figure S1.** Gamma distributions from which subordinate values (*h*) were drawn for individual subpopulations. Habitats were such that adding subordinates resulted in low productivity gains (mean *h* = 0.5; gamma function variables, *k* = 4 and *θ* = 0.125), medium (mean *h* = 1.5; *k* = 4 and *θ* = 0.3875), or high (mean *h* = 2.5; *k* = 8 and *θ* = 0.38). Dotted lines indicate where combinations of *x, y* and *p* values are possible such that increasing numbers of subordinates could do as well or better than being solitary.


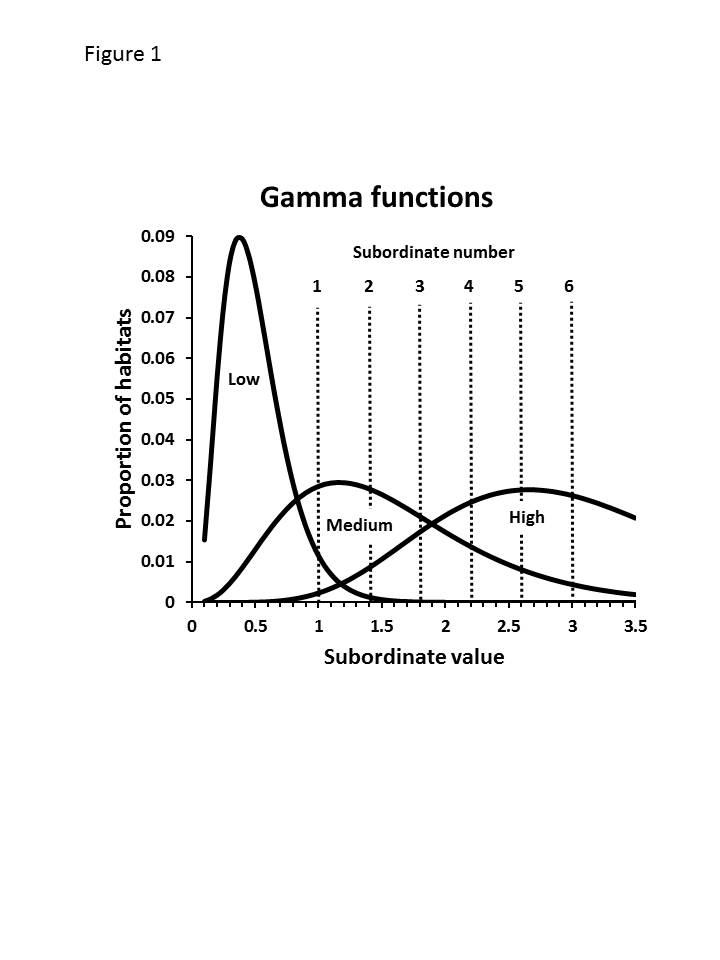


Model code:

!DEFINE NEEDED ARRAYS

DIM Animals(1,1,1,1)

DIM AnimalsNew(1,1,1,1)

DIM Migrants(1,1)

DIM GroupFit(1,1)

DIM SubFit(1)

DIM GroupSize(1,1)

DIM GammaArray(69,2)

DIM SitePicks(1)

DIM Frequencies(0:100,4,2)

LET Printon = 1 !TURNS ON FILE MAKING

LET Tanimals = 480 !TOTAL # OF ANIMALS

LET Subpops = 12 !# OF SUBPOPULATIONS

LET PopMax = Tanimals/Subpops !ANIMALS PER SUBPOPULATION

LET gmax = 5000 !# OF GENERATIONS PER SIMULATION RUN

LET gswitch = gmax * 0.5 !SWITCH POINT TO LINKED GENOMES AND HABITAT VARIANCE

LET smax = 32 !# OF SIMULATIONS

LET Manipulation = 1 !ON VERSUS OFF (0 VS 1)

!CONTINUOUS VARIABLES (BETWEEN 0 AND 1+)

!MUTATION RATE AND LARGEST MAGNITUDE OF MUTATION

LET mutationRateA = 0.02

LET mutation = 0.05

!CHANCE OF DYING IN ANY GENERATION (0 OR 0.1)

LET mortality = 0

!NUMBER OF CHOICES BETWEEN SITES (1 OR 3)

LET cmax = 1

!PROPORTION OF SUBORDINATES THAN WOULD BE OFFSPRING OF DOMINANT (0 TO 1)

LET Kin = 1

!DIMENSION NEEDED ARRAYS

MAT REDIM Subfit(Subpops)

MAT REDIM SitePicks(cmax)

MAT REDIM Migrants(Tanimals,6)

!FILE NAMING

LET AAA$ = "Base_"

IF Kin > 0 then

IF Kin = 1 then LET AAA$ = "Kin_" else LET AAA$ = "PKin_"

END IF

IF mortality > 0 then LET AAA$ = "Mort_"

IF cmax > 1 then LET AAA$ = "Choice_"

IF Manipulation = 1 then LET AAA$ = AAA$ & "C_"

RANDOMIZE

PRINT "Start"

FOR benefit = 0.5 to 2.501 step 1 !ADDED VALUE FOR 1ST SUBORDINATE

!FILE NAMING

LET BBB$ = "Low B_"

IF benefit > 1 then LET BBB$ = "Med B_"

IF benefit > 2 then LET BBB$ = "Hi B_"

FOR mixing = 1 to 3 !SETS PER GENERATION MIGRATION RATE BETWEEN SUBPOPULATIONS

IF mixing = 1 then

LET DDD$ = "Low M"

LET migrate = 1/Popmax

END IF

IF mixing = 2 then

LET DDD$ = "Med M"

LET migrate = 0.1

END IF

IF mixing = 3 then

LET DDD$ = "Hi M"

LET migrate = 0.5

END IF

FOR density = 1 to 3

IF density = 1 then !SETS RATIO OF ANIMALS TO AVAILABLE SITES

LET nests = Popmax

LET nmax = 6 !CAP TO MAX GROUP #

LET CCC$ = "1&1_"

END IF

IF density = 2 then

LET nests = Popmax/2

LET nmax = 8

LET CCC$ = "2&1_"

END IF

IF density = 3 then

LET nests = Popmax/5

LET nmax = 12

LET CCC$ = "5&1_"

END IF

!DIMENSION NEEDED ARRAYS

MAT REDIM Animals(Subpops,nests,nmax,10)

MAT REDIM AnimalsNew(Subpops,nests,nmax,10)

MAT REDIM GroupSize(Subpops,nests)

MAT REDIM GroupFit(Subpops,nmax)

!FILE PRINTING COMMANDS

LET WWW$ = AAA$ & BBB$ & CCC$ & DDD$

LET WWW2$ = "G_" & WWW$

PRINT WWW$, TIME$

IF PrintOn = 1 then

OPEN #4: name "s" & WWW$ & ".txt", create newold, access outin, organization text

SET #4: MARGIN 1000

PRINT #4: WWW$; ","; "Habitat"; ","; "Migrate"; ","; "Ratio"; ","; "SimCat"; ","; "Pop"; ",";

PRINT #4: "Dom"; ","; "SD"; ","; "Sub"; ",";

IF Manipulation = 1 then PRINT #4: "Sub*Cont"; ",";

PRINT #4: "SD"; ","; "Concede"; ","; "SD"; ",";

IF Manipulation = 1 then PRINT #4: "Control"; ","; "SD"; ",";

PRINT #4: "Dom Fit"; ","; "SD"; ","; "Sub Fit"; ","; "SD"; ","; "% of Dom"; ","; "SD"; ","; "Realized"; ","; "SD"

OPEN #5: name "s" & WWW2$ & ".txt", create newold, access outin, organization text

SET #5: MARGIN 1000

PRINT #5: "Proportion"; ","; "Dominant"; ","; "Dominant"; ","; "Subordinate"; ","; "Subordinate"; ","; "Concede"; ","; "Concede"; ",";

IF Manipulation = 1 then PRINT #5: "Control"; ","; "Control"

END IF

MAT Frequencies = 0

LET Ddead = 0

LET Sdead = 0

LET Tbenefit = 0

LET deficit = 0

LET Bnum = 0

LET dcountX = 0

LET scountX = 0

LET ccountX = 0

LET dvalueX = 1

LET svalueX = 1

LET cvalueX = 1

LET mult = (-1)

LET simCat = 1

FOR sim = 1 to smax

LET dcountX = dcountX + 1

LET scountX = scountX + 1

LET ccountX = ccountX + 1

IF dcountX > smax/2 then

LET dvalueX = dvalueX * mult

LET dcountX = 1

END IF

IF scountX > smax/4 then

LET svalueX = svalueX * mult

LET scountX = 1

END IF

IF ccountX > smax/8 then

LET cvalueX = cvalueX * mult

LET ccountX = 1

LET simCat = simCat + 1

END IF

!FILL IN INITIAL POPULATIONS WITH ANIMALS OF GIVEN GENOTYPES

MAT Animals = 0

MAT GroupSize = 0

FOR i = 1 to Subpops

FOR j = 1 to PopMax

LET check = 0

DO while check = 0

LET nest = int(rnd*nests) + 1

IF GroupSize(i,nest) < nmax THEN

LET check = 1

LET ID = GroupSize(i,nest) + 1

LET GroupSize(i,nest) = ID !CHOOSE A SITE FOR THE NEXT ANIMAL

LET Animals(i,nest,ID,1) = nest

LET Animals(i,nest,ID,2) = 0 !LOCATION FOR REALIZED FITNESS OF GROUP

IF dvalueX > 0 then LET vmin = 0 else LET vmin = 0.3

LET Animals(i,nest,ID,3) = vmin + rnd*0.2 !ANIMAL STARTS WITH EITHER LOW OR HIGH DOMINANT COMPETITIVENESS LEVEL

IF svalueX > 0 then LET vmin = 0 else LET vmin = 0.3

LET Animals(i,nest,ID,4) = vmin + rnd*0.2 !ANIMAL STARTS WITH EITHER LOW OR HIGH SUBORDINATE COMPETITIVENESS LEVEL

IF cvalueX > 0 then LET vmin = 0 else LET vmin = 0.5

LET Animals(i,nest,ID,5) = vmin + rnd*0.3 !ANIMAL STARTS WITH EITHER LOW OR HIGH DOMINANT CONCESSION LEVEL

IF Manipulation = 0 then LET Animals(i,nest,ID,6) = 1 else LET Animals(i,nest,ID,6) = rnd !SET CONTROL GENOTYPE IF NEEDED (NO CONTROL = 1)

LET Animals(i,nest,ID,7) = 0 !LOCATION FOR REALIZED FITNESS OF THIS INDIVIDUAL

LET Animals(i,nest,ID,8) = 0 !LOCATION FOR RELATIVE REALIZED FITNESS OF INDIVIDUAL IN SUBPOPULATION

LET Animals(i,nest,ID,9) = 0 !LOCATION FOR RELATIVE REALIZED FITNESS OF INDIVIDUAL IN ENTIRE POPULATION

LET Animals(i,nest,ID,10) = 0 !DOMINANT OR SUBORDINATE?

END IF

LOOP

NEXT j

NEXT i

FOR gen = 0 to gmax

IF gen = 0 or gen = gswitch then CALL SetFit

LET mutationRate = mutationRateA

MAT SubFit = 0

LET Tfitness = 0

!SET VALUES FROM dominant AND subordinate GENOTYPES FOR COMPETITION. CONCESSION AND CONTROL

FOR i = 1 to Subpops

FOR j = 1 to nests

LET Anum = 0

LET domC = 0

LET domX = 0

FOR k = 1 to nmax

IF Animals(i,j,k,1) > 0 then

LET anum = anum + 1

IF anum = 1 then !DOMINANT-EXPRESSED TRAITS

LET domX = Animals(i,j,k,3)

LET domC = Animals(i,j,k,5)

LET Dcontrol = Animals(i,j,k,6)

LET subtotY = 0

LET submaxY = 0

ELSE

!SUBORDINATE-EXPRESSES TRAIT

LET subtotY = subtotY + Animals(i,j,k,4)

IF Animals(i,j,k,4) > submaxY then LET submaxY = Animals(i,j,k,4) !Y VALUE FOR COMPETITION COST

END IF

ELSE

LET k = nmax

END IF

NEXT k

IF anum > 0 then

FOR k = 1 to anum

IF anum = 1 then

!SOLITARY NEST

!DOES THE ANIMAL DIE IN THIS GENERATION?

LET dead = 0

LET Animals(i,j,k,2) = 0

IF rnd >= mortality then !NO

LET Animals(i,j,k,7) = 1 !SOLITARY FITNESS

LET RealFit = 0 !NO SUBS = NO ADDED VALUE

LET alive = 1

ELSE !YES

LET Animals(i,j,k,7) = 0

LET Animals(i,j,k,10) = 1

LET alive = 0

END IF

ELSE

LET compete = 1 - domX - Dcontrol * submaxY !PROPORTION OF ADDED VALUE NOT LOST TO COMPETITION

IF compete < 0 then LET compete = 0

LET RealFit = (GroupFit(i,anum) - 1) * compete !PROPORTION OF TOTAL ADDED VALUE THAT REMAINS (REALIZED) AFTER COMPETITION

LET Animals(i,j,k,2) = compete

IF k = 1 then

IF rnd >= mortality then !ORIGINAL DOMINANT ALIVE

!DOMINANT'S FITNESS

IF (Dcontrol*subtotY) > 0 then

LET Animals(i,j,k,7) = 1 + Realfit * (1 - domC) * domX / (domX + Dcontrol * subtotY)

ELSE

LET Animals(i,j,k,7) = 1 + Realfit * (1 - domC)

END IF

LET alive = 1

LET dead = 0

LET anum1 = 1

ELSE !ORIGINAL DOMINANT IS DEAD

LET Animals(i,j,k,7) = 0

LET Animals(i,j,k,10) = 1

LET dead = 1

LET alive = 0

LET anum1 = 0

END IF

ELSE

!ORIGINAL SUBORDINATE IS ALIVE

!SUB'S PROPORTION OF DOMINANT-CONCEDED REPRODUCTION

IF subtotY > 0 then

LET SubConcede = Realfit * domC * Animals(i,j,k,4) / subtotY

ELSE

LET SubConcede = Realfit * domC / (anum - 1)

END IF

IF (subtotY*Dcontrol) > 0 then

!SUB'S PROPORTION OF CONTESTED REPRODUCTION

LET SubComp = Realfit * (1 - domC) * Animals(i,j,k,4) * Dcontrol / (subtotY * Dcontrol + domX)

ELSE

LET SubComp = 0

END IF

!SUBORDINATES'S SUMMED FITNESS

LET Animals(i,j,k,7) = SubConcede + SubComp

IF rnd < mortality then !ORIGINAL SUBORDINATE IS DEAD

LET Animals(i,j,k,7) = 0

LET Animals(i,j,k,10) = 1

LET dead = 1

ELSE !STILL ALIVE

LET alive = 1

LET anum1 = anum1 + 1

END IF

END IF

END IF

NEXT k

!ORIGINALLY MORE THAN ONE IN GROUP AND AT LEAST ONE IS DEAD

IF dead = 1 and alive = 1 then

IF anum1 > 1 then !MORE THAN ONE GROUP MEMBER STILL ALIVE

CALL Redistribute

ELSE !ONLY ONE STILL ALIVE

FOR k = 1 to anum

IF Animals(i,j,k,10) = 1 then !DEAD GROUP MEMBER

LET Animals(i,j,k,7) = 0

ELSE !LIVING GROUP MEMBER

LET Animals(i,j,k,7) = GroupFit(i,anum)

END IF

NEXT k

END IF

END IF

!COLLECTING VALUES FOR CALCULATING MEANS & SD'S

IF alive = 1 then

LET SubFit(i) = Subfit(i) + RealFit + 1

LET Tfitness = Tfitness + RealFit + 1

END IF

END IF

NEXT j

NEXT i

!PUT ALL LIVE ANIMALS INTO POTENTIAL WITHIN-SUBPOPULATION AND MIGRANT-PRODUCING POOL

LET oldfit = 0

MAT Migrants = 0

LET count = 0

FOR i = 1 to Subpops

LET oldfit1 = 0

FOR j = 1 to nests

FOR k = 1 to nmax

!ANIMAL'S RELATIVE FITNESS WITHIN SUBPOPULATION

LET Animals(i,j,k,8) = oldfit1 + Animals(i,j,k,7) / Subfit(i)

!ANIMAL'S RELATIVE FITNESS FOR PRODUCING MIGRANTS

IF Animals(i,j,k,7) > 0 then

LET oldfit1 = Animals(i,j,k,8)

LET count = count + 1

LET Migrants(count,1) = oldfit + Animals(i,j,k,7) / Tfitness

FOR m = 3 to 6 !RECORD GENOTYPES

LET Migrants(count,m) = Animals(i,j,k,m)

NEXT m

LET oldfit = Migrants(count,1)

END IF

NEXT k

NEXT j

NEXT i

!CREATE COUNTER FOR RAPID SEARCH OF MIGRANT POOL

LET Ccount = count/4 - int(count/4)

IF Ccount = 0.25 then LET count = count + 3

IF Ccount = 0.5 then LET count = count + 2

IF Ccount = 0.75 then LET count = count + 1

!CHOOSE NEXT GENERATION OF ANIMALS

MAT AnimalsNew = 0

FOR i = 1 to Subpops

FOR j = 1 to Popmax

IF rnd < migrate then !CHOICE IS A MIGRANT

FOR k = 1 to kmax

!SEARCH MIGRANT ARRAY FOR PARENT

!LOCATE AREA IN ARRAY TO SEARCH

LET ipick = rnd

IF ipick < Migrants(count/2,1) then

IF ipick < Migrants(count/4,1) then

LET iimin = 1

ELSE

LET iimin = count/4

END IF

ELSE

IF ipick < Migrants(3*count/4,1) then

LET iimin = count/2

ELSE

LET iimin = 3*count/4

END IF

END IF

FOR ii = iimin to Tanimals

LET aaa = migrants(ii,1)

IF aaa >= ipick then

IF k = 1 then !PARENT #1

LET dom = migrants(ii,3)

LET sub = migrants(ii,4)

LET concede = migrants(ii,5)

LET control = migrants(ii,6)

ELSE !PARENT #2 (IF ALLELES NOT LINKED)

IF rnd < 0.5 then LET dom = migrants(ii,3)

IF rnd < 0.5 then LET sub = migrants(ii,4)

IF rnd < 0.5 then LET concede = migrants(ii,5)

IF rnd < 0.5 then LET control = migrants(ii,6)

END IF

LET ii = Tanimals

CALL PreMutate !ARE ANY OF THE ALLELES MUTATED?

END IF

NEXT ii

NEXT k

ELSE !CHOICE COMES FROM WITHIN THE SUBPOPULATION

FOR k = 1 to kmax

LET ipick = rnd

FOR jj = 1 to nests

FOR kk = 1 to nmax

LET aaa = animals(i,jj,kk,8)

IF aaa >= ipick then

IF k = 1 then !PARENT #1

LET dom = animals(i,jj,kk,3)

LET sub = animals(i,jj,kk,4)

LET concede = animals(i,jj,kk,5)

LET control = animals(i,jj,kk,6)

ELSE !PARENT #2 (IF ALLELES NOT LINKED)

IF rnd < 0.5 then LET dom = animals(i,jj,kk,3)

IF rnd < 0.5 then LET sub = animals(i,jj,kk,4)

IF rnd < 0.5 then LET concede = animals(i,jj,kk,5)

IF rnd < 0.5 then LET control = animals(i,jj,kk,6)

END IF

LET jj = nests

LET kk = nmax

CALL PreMutate !ARE ANY OF THE ALLELES MUTATED?

END IF

NEXT kk

NEXT jj

NEXT k

END IF

!CHOOSE BEST GROUP FROM 1 OR MORE CHOICES

LET bestnest = -999

MAT SitePicks = 0

FOR choices = 1 to cmax

LET pick = 0

DO while pick = 0

LET nest = int(rnd * nests) + 1 !NEST CHOICE

!MAKES SURE CHOICES ARE NOT DUPLICATES

LET nogo = 0

FOR cc = 1 to cmax

IF nest = SitePicks(cc) then LET nogo = 1

NEXT cc

IF nogo = 0 then

FOR cc = 1 to cmax

IF SitePicks(cc) = 0 then LET SitePicks(cc) = nest

NEXT cc

FOR k = 1 to nmax

IF AnimalsNew(i,nest,k,1) = 0 then

LET cnum = k

CALL Best_Choice

!IS NEW CHOICE BETTER THAN ANY PREVIOUS CHOICE?

IF fitness > bestnest then

LET optnest = nest

LET optcnum = cnum

LET bestnest = fitness

END IF

LET k = nmax

LET pick = 1

END IF

NEXT k

END IF

LOOP

NEXT choices

!RECORD GENOTYPE OF ANIMAL AT THE NEST CHOSEN AND WHICH GROUP # MEMBER IT IS

LET AnimalsNew(i,optnest,optcnum,1) = optnest

LET AnimalsNew(i,optnest,optcnum,3) = dom

LET AnimalsNew(i,optnest,optcnum,4) = sub

LET AnimalsNew(i,optnest,optcnum,5) = concede

LET AnimalsNew(i,optnest,optcnum,6) = control

NEXT j

!REPLACE SUBORDINATES WITH KIN IF NEEDED TO

IF kin > 0 then CALL KinGroup

IF gen = gmax then CALL Outputs

NEXT i

MAT Animals = AnimalsNew

NEXT gen

NEXT sim

!PRINTING COMMANDS

IF Printon = 1 then

IF Manipulation = 1 then LET jmaxX = 4 else LET jmaxX = 3

LET totals = 0

FOR i = 0 to 100

LET totals = totals + Frequencies(i,1,x)

NEXT i

FOR i = 0 to 100

PRINT #5: i/100; ",";

FOR j = 1 to jmaxX

LET doms = Frequencies(i,j,x)/totals

PRINT #5: Frequencies(i,j,x); ","; round(doms,4); ",";

NEXT j

PRINT #5

NEXT i

CLOSE #4

CLOSE #5

END IF

NEXT density

NEXT mixing

NEXT benefit

!SUBROUTINES**********************************************

SUB Outputs

!PRINTING COMMANDS

IF gen = gswitch then LET x = 1 else LET x = 2

LET Ydom = 0

LET Ysub = 0

LET Yconcede = 0

LET Ycontrol = 0

LET Ydomfitness = 0

LET Ysubfitness = 0

LET Ydom2 = 0

LET Ysub2 = 0

LET Yconcede2 = 0

LET Ycontrol2 = 0

LET Ydomfitness2 = 0

LET Ysubfitness2 = 0

LET domnum = 0

LET subnum = 0

LET Yrealized = 0

LET Yrealized2 = 0

LET YmeanFit = 0

LET YmeanFit2 = 0

LET gnum = 0

FOR j = 1 to nests

LET anum = 0

FOR k = 1 to nmax

IF Animals(i,j,k,1) > 0 then

LET anum = anum + 1 !ORIGINAL GROUP SIZE

ELSE

LET k = nmax

END IF

NEXT k

IF anum > 1 then

LET realized = Animals(i,j,1,2)

LET Yrealized = Yrealized + realized

LET Yrealized2 = Yrealized2 + realized^2

LET gnum = gnum + 1 !# OF GROUPS WITH > 1

END IF

LET Tsub = 0

LET domfitness = 0

LET subfitness = 0

LET subAlive = 0

FOR k = 1 to nmax

IF Animals(i,j,k,1) > 0 then

LET dommean = Animals(i,j,k,3)

LET submean = Animals(i,j,k,4)

LET concedemean = Animals(i,j,k,5)

LET controlmean = Animals(i,j,k,6)

LET Ydom = Ydom + dommean

LET Ydom2 = Ydom2 + dommean^2

LET Ysub = Ysub + submean

LET Ysub2 = Ysub2 + submean^2

LET Yconcede = Yconcede + concedemean

LET Yconcede2 = Yconcede2 + concedemean^2

LET Ycontrol = Ycontrol + controlmean

LET Ycontrol2 = Ycontrol2 + controlmean^2

IF k = 1 and anum > 1 THEN

IF Animals(i,j,k,10) = 0 then

LET domfitness = Animals(i,j,k,7)

LET Ydomfitness = Ydomfitness + domfitness

LET Ydomfitness2 = Ydomfitness2 + domfitness^2

LET domnum = domnum + 1 !# OF TOTAL SURVIVING DOMINANTS

LET domAlive = 1 !DOMINANT IS ALIVE

ELSE

LET domAlive = 0

LET ddead = ddead + 1

END IF

ELSE

IF anum > 1 then

IF Animals(i,j,k,10) = 0 then

LET subfitness = Animals(i,j,k,7)

LET Tsub = Tsub + subfitness

LET Ysubfitness = Ysubfitness + subfitness

LET Ysubfitness2 = Ysubfitness2 + subfitness^2

LET subnum = subnum + 1 !# OF TOTAL SURVIVING SUBORDINATES

LET subAlive = subAlive + 1 !AT LEAST ONE SUBORDINATE AT SITE IS ALIVE

ELSE

LET sdead = sdead + 1

END IF

END IF

END IF

LET doms = round(Animals(i,j,k,3),2)*100

LET frequencies(doms,1,x) = frequencies(doms,1,x) + 1

LET doms = round(Animals(i,j,k,4),2)*100

LET frequencies(doms,2,x) = frequencies(doms,2,x) + 1

LET doms = round(Animals(i,j,k,5),2)*100

LET frequencies(doms,3,x) = frequencies(doms,3,x) + 1

IF Manipulation = 1 then

LET doms = round(Animals(i,j,k,6),2)*100

LET frequencies(doms,4,x) = frequencies(doms,4,x) + 1

END IF

ELSE

LET k = nmax

IF anum > 1 then

IF domAlive = 1 or subAlive > 0 then

LET YMeanFit = YMeanFit + Tsub / (Tsub + domfitness)

LET YMeanFit2 = YMeanFit2 + (Tsub / (Tsub + domfitness))^2

END IF

END IF

END IF

NEXT k

NEXT j

LET YdomSD = (((Ydom2 - PopMax * (Ydom/PopMax)^2) / (PopMax - 1)))

LET YsubSD = (((Ysub2 - PopMax * (Ysub/PopMax)^2) / (PopMax - 1)))

LET YconcedeSD = (((Yconcede2 - PopMax * (Yconcede/PopMax)^2) / (PopMax - 1)))

LET YcontrolSD = (((Ycontrol2 - PopMax * (Ycontrol/PopMax)^2) / (PopMax - 1)))

IF YdomSD > 0 then LET YdomSD = round(YdomSD^0.5,5)

IF YsubSD > 0 then LET YsubSD = round(YsubSD^0.5,5)

IF YconcedeSD > 0 then LET YconcedeSD = round(YconcedeSD^0.5,5)

IF YcontrolSD > 0 then LET YcontrolSD = round(YcontrolSD^0.5,5)

IF domnum > 0 then

IF domnum > 1 then

LET YdomfitnessSD = (((Ydomfitness2 - domnum * (Ydomfitness/domnum)^2) / (domnum - 1)))

ELSE

LET YdomfitnessSD = 0

END IF

ELSE

LET domnum = 1

END IF

IF subnum > 0 then

IF subnum > 1 then

LET YsubfitnessSD = (((Ysubfitness2 - subnum * (Ysubfitness/subnum)^2) / (subnum - 1)))

ELSE

LET YsubfitnessSD = 0

END IF

ELSE

LET subnum = 1

END IF

IF gnum > 0 then

IF gnum > 1 then

LET YMeanFitSD = (((YMeanFit2 - gnum * (YMeanFit/gnum)^2) / (gnum - 1)))

LET YrealizedSD = (((Yrealized2 - gnum * (Yrealized/gnum)^2) / (gnum - 1)))

ELSE

LET YMeanFitSD = 0

LET YrealizedSD = 0

END IF

ELSE

LET gnum = 1

END IF

IF YdomfitnessSD > 0 then LET YdomfitnessSD = round(YdomfitnessSD^0.5,5)

IF YsubfitnessSD > 0 then LET YsubfitnessSD = round(YsubfitnessSD^0.5,5)

IF YMeanFitSD > 0 then LET YMeanFitSD = round(YMeanFitSD^0.5,5)

IF YrealizedSD > 0 then LET YrealizedSD = round(YrealizedSD^0.5,5)

IF printon = 1 then

PRINT #4: WWW$; ","; benefit; ","; migrate; ","; CCC$; ","; simCat; ","; i; ",";

PRINT #4: round(Ydom/popmax,5); ","; YdomSD; ","; round(Ysub/popmax,5); ",";

IF Manipulation = 1 then PRINT #4: round((Ysub/popmax)*(Ycontrol/popmax),5); ",";

PRINT #4: YsubSD; ","; round(Yconcede/popmax,5); ","; YconcedeSD; ",";

IF Manipulation = 1 then PRINT #4: round(Ycontrol/popmax,5); ","; YcontrolSD; ",";

PRINT #4: round(Ydomfitness/domnum,5); ","; YdomfitnessSD; ","; round(Ysubfitness/subnum,5); ","; YsubfitnessSD; ",";

PRINT #4: round(YMeanFit/gnum,5); ","; YMeanFitSD; ","; round(Yrealized/gnum,5); ","; YrealizedSD

END IF

END SUB

SUB SetFit

!SETS IF ALLELES ARE LINKED (YES = 1)

IF gen = 0 then LET kmax = 2 ELSE LET kmax = 1

!FITNESS FUNCTION FOR GROUP SIZES

!AT gen = gswitch HABITATS BECOME VARIABLE FOR SUB QUALITY ACROSS SUBPOPULATIONS

MAT groupFit = 0

FOR ii = 1 to SubPops !SUBPOPULATIONS VARY IN THE ADDED PRODUCTIVITY THAT SUBORDINATES PROVIDE

LET GroupFit(ii,1) = 1

IF gen = gswitch then

LET Bnum = Bnum + 1

IF deficit = 0 then

CALL Habitat

LET Tbenefit = Tbenefit + benMean

LET deficit = benefit * Bnum - Tbenefit

ELSE

!IF RUNNING AVERAGE IS ABOVE OR BELOW SET BENEFIT MEAN, WILL CHOOSE VALUES TO MOVE BACK TO MEAN

LET benMean = benefit * Bnum - Tbenefit

IF benMean < 0.1 then

LET deficit = 0.1 - benMean

LET benMean = 0.1

ELSE

LET deficit = 0

END IF

LET Tbenefit = Tbenefit + benMean

END IF

ELSE

LET benMean = benefit

END IF

!PLACES MAXIMUM POTENTIAL GAIN FOR EACH ADDITIONAL SUBORDINATE INTO AN ARRAY

FOR jj = 2 to nmax

LET GroupFit(ii,jj) = GroupFit(ii,jj-1) + benMean / 2^(jj-2)

NEXT jj

NEXT ii

IF gen = gswitch then PRINT sim, round(benMean,5), round(Tbenefit/Bnum,5) !TRACK RUNNING AVERAGE

END SUB

SUB Habitat

!DRAWS benefit RANDOMLY FROM A GAMMA DISTRIBUTION

LET kay = 4

LET theta = 0.128

IF benefit > 1 then

LET kay = 4

LET theta = 0.3875

END IF

IF benefit > 2 then

LET kay = 8

LET theta = 0.38

END IF

LET KFact = 1

FOR iii = 1 to kay

LET KFact = KFact * iii

NEXT iii

LET KFact = 1 / (KFact * theta^kay)

LET gammaStep = 0

LET gammaTotal = 0

MAT GammaArray = 0

FOR xx = 0.1 to 3.501 step 0.1

LET gammaStep = gammaStep + 1

LET gamma = KFact * xx^(kay-1) * exp((0-xx)/theta)

LET gammaArray(gammaStep,1) = xx

LET gammaArray(gammaStep,2) = gamma

LET gammaTotal = gammaTotal + gamma

NEXT xx

LET gammaArray(1,2) = gammaArray(1,2)/gammaTotal

FOR iii = 2 to gammaStep-1

LET gammaArray(iii,2) = gammaArray(iii-1,2) + gammaArray(iii,2)/gammaTotal

NEXT iii

LET gammaArray(gammaStep,2) = 1

LET Bpick = rnd

FOR iii = 1 to gammaStep

IF Bpick <= gammaArray(iii,2) then

LET benMean = gammaArray(iii,1) !CHOSEN h VALUE FOR THIS SUBPOPULATION

LET iii = gammaStep

END IF

NEXT iii

END SUB

SUB PreMutate

!CHANGE ALLELE VALUE IF MUTATED

LET xxx = dom

CALL Mutate

LET dom = xxx

LET xxx = sub

CALL Mutate

LET sub = xxx

LET xxx = concede

CALL Mutate

LET concede = xxx

IF Manipulation = 1 then

LET xxx = control

CALL Mutate

LET control = xxx

ELSE

LET control = 1 !NO CONTROL OVER COMPETITION

END IF

END SUB

SUB Mutate

!MUTATE EACH ALLELE BETWEEN +/- 0.5 IN MAGNITUDE

IF RND < mutationRate then

IF RND < 0.5 then LET xxx = xxx + mutation * rnd else LET xxx = xxx - mutation * rnd

!UPPER AND LOWER BOUNDS OF ALLELE VALUES

IF xxx > 1 then LET xxx = 1

IF xxx < 0 then LET xxx = 0

END IF

END SUB

SUB KinGroup

FOR jj = 1 to nests

IF AnimalsNew(i,jj,1,1) > 0 then

FOR kk = 1 to nmax

IF kk = 1 then

!DOMINANT = PARENT OF SUBORDINATE

LET dom1 = AnimalsNew(i,jj,kk,3)

LET sub1 = AnimalsNew(i,jj,kk,4)

LET concede1 = AnimalsNew(i,jj,kk,5)

LET control1 = AnimalsNew(i,jj,kk,6)

!PICK 2ND PARENT FROM SAME SUBPOPULATION

LET ipick = rnd

FOR iii = 1 to nests

FOR jjj = 1 to nmax

LET aaa = Animals(i,iii,jjj,8)

IF aaa >= ipick then

LET dom4 = Animals(i,iii,jjj,3)

LET sub4 = Animals(i,iii,jjj,4)

LET concede4 = Animals(i,iii,jjj,5)

LET control4 = Animals(i,iii,jjj,6)

LET iii = nests

LET jjj = nmax

END IF

NEXT jjj

NEXT iii

ELSE

IF AnimalsNew(i,jj,kk,1) > 0 then

!REPLACE CURRENT SUBORDINATE WITH OFFSPRING

IF rnd < kin then

IF rnd < 0.5 then LET AnimalsNew(i,jj,kk,3) = dom1 else LET AnimalsNew(i,jj,kk,3) = dom4

IF rnd < 0.5 then LET AnimalsNew(i,jj,kk,4) = sub1 else LET AnimalsNew(i,jj,kk,4) = sub4

IF rnd < 0.5 then LET AnimalsNew(i,jj,kk,5) = concede1 else LET AnimalsNew(i,jj,kk,5) = concede4

IF rnd < 0.5 then LET AnimalsNew(i,jj,kk,6) = control1 else LET AnimalsNew(i,jj,kk,6) = control4

END IF

ELSE

LET kk = nmax

END IF

END IF

NEXT kk

END IF

NEXT jj

END SUB

SUB Redistribute

!RECALCULATE LOSS TO COMPETITION WITH MORTALITY OF GROUP MEMBER

LET dominant = 0

FOR kk = 1 to anum

IF Animals(i,j,kk,10) = 0 then !IS THE ANIMAL ALIVE?

IF dominant = 0 then !IF 1ST LIVING ANIMAL = DOMINANT

LET dominant = 1

LET domX = Animals(i,j,kk,3)

LET domC = Animals(i,j,kk,5)

LET Dcontrol = Animals(i,j,kk,6)

LET subtotY = 0

LET submaxY = 0

LET anum1 = 1

ELSE

!SUBORDINATE VALUES

LET subtotY = subtotY + Animals(i,j,kk,4)

IF Animals(i,j,kk,4) > submaxY then LET submaxY = Animals(i,j,kk,4)

LET anum1 = anum1 + 1

END IF

END IF

NEXT kk

!CALCULATE FITNESSES FOR LIVING ANIMALS (= REDISTRIBUTING ADDED VALUE OF DEAD MEMBERS)

LET RealFit = (GroupFit(i,anum) - 1) * compete

LET dominant = 0

FOR kk = 1 to anum

LET Animals(i,j,kk,2) = compete

IF Animals(i,j,kk,10) = 0 then

IF dominant = 0 then

LET dominant = 1

IF (Dcontrol*subtotY) > 0 then

LET Animals(i,j,kk,7) = 1 + Realfit * (1 - domC) * domX / (domX + Dcontrol * subtotY)

ELSE

LET Animals(i,j,kk,7) = 1 + Realfit * (1 - domC)

END IF

ELSE

IF subtotY > 0 then

LET SubConcede = Realfit * domC * Animals(i,j,kk,4) / subtotY

ELSE

LET SubConcede = Realfit * domC / (anum1 - 1)

END IF

IF (subtotY*Dcontrol) > 0 then

LET SubComp = Realfit * (1 - domC) * Animals(i,j,kk,4) * Dcontrol / (subtotY * Dcontrol + domX)

ELSE

LET SubComp = 0

END IF

LET Animals(i,j,kk,7) = SubConcede + SubComp

END IF

END IF

NEXT kk

END SUB

SUB Best_Choice

IF cnum = 1 then !WOULD BE 1ST INDIVIDUAL AT THAT SITE

LET fitness = 1

ELSE

!CALCULATE EXPECTED FITNESS AT SITE AS A SUBORDINATE

FOR kk = 1 to cnum

IF kk = 1 then

LET domX = AnimalsNew(i,nest,kk,3)

LET domC = AnimalsNew(i,nest,kk,5)

LET Dcontrol = AnimalsNew(i,nest,kk,6)

LET subtotY = 0

LET submaxY = 0

ELSE

IF kk < cnum then

LET subtotY = subtotY + AnimalsNew(i,nest,kk,4)

IF AnimalsNew(i,nest,kk,4) > submaxY then LET submaxY = AnimalsNew(i,nest,kk,4)

ELSE

LET subtotY = subtotY + sub

IF sub > submaxY then LET submaxY = sub

END IF

END IF

NEXT kk

LET compete = 1 - domX - Dcontrol * submaxY

IF compete < 0 then LET compete = 0

LET RealFit = (GroupFit(i,cnum) - 1) * compete

IF subtotY > 0 then

LET SubConcede = Realfit * domC * sub / subtotY

ELSE

LET SubConcede = Realfit * domC / (cnum - 1)

END IF

IF (subtotY*Dcontrol) > 0 then

LET SubComp = Realfit * (1 - domC) * sub * Dcontrol / (subtotY * Dcontrol + domX)

ELSE

LET SubComp = 0

END IF

LET fitness = SubConcede + SubComp

END IF

END SUB

PRINT "Finished"

END
